# A Comparative Health Risk Assessment of Electronic Cigarettes and Conventional Cigarettes

**DOI:** 10.3390/ijerph14040382

**Published:** 2017-04-05

**Authors:** Jinsong Chen, Chris Bullen, Kim Dirks

**Affiliations:** 1 National Institute for Health Innovation, School of Population Health, Faculty of Medical and Health Science, University of Auckland, Auckland 1010, New Zealand; jche403@aucklanduni.ac.nz; 2 School of Population Health, Faculty of Medical and Health Science, University of Auckland, Auckland 1010, New Zealand; k.dirks@auckland.ac.nz

**Keywords:** tobacco control, electronic cigarettes, toxicology, comparative risk, risk assessment

## Abstract

*Background*: Although some studies have identified hazardous substances in electronic cigarette (EC) liquids and emissions, there is limited information about the health risks of using ECs. *Methods*: In this study, the U.S. Environmental Protection Agency (EPA) health risk assessment model and findings of a literature review were used to determine and profile hazards. Focus was put on the toxicants reported in the literature on conventional cigarette (CC) smoke that most strongly associated with adverse health effects. To evaluate their health risks, dose-response relationships and standard-use conditions were used to estimate average hazard exposures and to calculate the overall health risks of ECs and CCs, benchmarked against international guideline levels for each hazard. *Results*: Four hazards (acrolein, diethylene glycol, propylene glycol and cadmium) reported in EC emissions and seven hazards (acetaldehyde, acrolein, formaldehyde, cadmium, CO, 4-(methylnitrosamino)-1-(3-pyridyl)-1-butanone (NNK), *N*′-nitrosonornicotine (NNN)) reported in CC emissions had maximum exposure levels higher than the guideline levels. Two hazards (acrolein, propylene glycol) in EC emissions and five hazards (acetaldehyde, acrolein, formaldehyde, cadmium, NNN) in CC emissions had average exposure levels higher than the guideline levels. *Conclusions*: Based on the conditions of use, ECs should be a safer nicotine-delivery product than CCs.

## 1. Introduction

Over the past few years, electronic cigarettes (ECs) have become one of the most preferred alternatives to conventional cigarettes (CCs) [1,2]. Their appearance, taste and use characteristics make them popular among smokers [2]. A survey carried out in 2014 in the United States (U.S.) found that 12.6% of adults had ever tried ECs, and almost half of current smokers (47.6%) and more than half of ex-smokers (55.4%) had ever tried ECs [3]. Another survey carried out in the UK in 2016 found that 61.1% of current adult smokers as well as 18.8% of ex-smokers and non-smokers had tried ECs [4]. ECs are attractive to smokers, especially among those who want to reduce their harm from smoking tobacco, those who want to quit smoking, as well as ex-smokers. Based on a survey done on March 2016, 29% of U.S. smokers tried ECs to quit smoking within the previous 6 months [5]. EC sales in the U.S. reached US $2.875 million in 2015, more than 1.5 times the sales levels reached in 2014 (US $1.70 million) [5].

In 2015, Public Health England and other UK medical bodies estimated that ECs are 95% less harmful than CCs [6,7]. This figure was based on research done in 2014 by the Independent Scientific Committee on Drugs using a Multi Criteria Decision Analysis (MCDA) model to estimate the relative harm of using different types of nicotine-containing products [6]. In this study, an International Expert Panel defined 14 harm criteria and scored all identified nicotine-delivery products on each criterion about their average harm (to both users and to others) using a scale of zero (no harm) to 100 (most harmful) [6]. On this basis, CCs emerged as the most harmful nicotine-containing product (scoring 100), while ECs were judged to be far less harmful (scoring 4) [6]. 

Despite this, most other health organisations have been more cautious in their public statements on the safety of ECs. For example, the U.S. Food and Drug Administration (U.S. FDA) reported that the potential health risks of using ECs are unclear [8]. At the same time, another study done by the U.S. Centers for Disease Control and Prevention (U.S. CDC) stated that emissions from ECs can contain low levels of potentially harmful constituents and pose health risk, while poorly characterised, heat-induced partial decomposition products of a highly complex tobacco extract in propylene glycol or glycerol may be harmful to an individual’s health [9]. 

Such differences in views exist in part because of the limited information available with which to quantify and compare the risk levels to health of using ECs relative to CCs. To our knowledge, no study has attempted to build a risk assessment model to evaluate the health risks of using ECs. We aimed to answer the questions: “Are ECs safe to use?” and “Are ECs safer to use than CCs”? A key point is that while ECs may not be “safe”, they may be “safer” than CCs, and therefore have potential, if used as CC substitutes, to significantly reduce, but not eliminate, harm, assuming the same consumer behaviour regarding use. We attempted to address these questions using a comparative risk assessment model to evaluate the human health risks of using ECs, with the health risk of smoking. 

## 2. Methods 

### 2.1. Methodology

For this study, the U.S. Environmental Protection Agency (EPA) human health risk assessment methodology was followed. This approach focuses on estimating the risk of certain behaviours/exposures of a specific population by identifying uncertainties, monitoring exposure to specific hazards, and evaluating the characteristics of hazards [10]. The assessment consists of five steps: scope planning, hazard identification, dose-response assessment, exposure assessment, and risk characterisation [11], in this case all based on pre-existing literature. We then carried out a risk comparison between ECs and CCs for a target population, estimating the relative safety and benchmarking the hazard exposure levels in EC and CC emissions in relation to recognised safe exposure levels (i.e., estimating the absolute safety).

### 2.2. Literature Review

A literature review was carried out using the PubMed database with search dates restricted to January 2005 when the first EC articles were published to June 2015. The primary search terms were: “electronic cigarette(s)” OR e-cig* OR “electronic nicotine delivery system(s)”. The secondary search terms were “chemical hazard(s)” OR carcinogen* OR nicotine; “animal experiment(s)” OR cytotoxic* OR “in vitro culturing”; “personal view(s)” OR perception(s) OR “clinical effect(s)” OR “acute effect(s)”. These were linked with the primary search terms with the Boolean operator “AND”.

The articles identified based on these search terms were then screened based on publication date, language (English language only), article type (clinical trial, journal article and research reports were included), as well as availability and appropriateness. Fifty articles were eventually selected and another 46 articles (either organisational reports or recommended articles from relevant literature reviews) were identified, giving a total of 96 research articles. From all identified articles, 42 were linked to chemical studies, 10 to toxicological studies, 40 to human studies, and 9 to non-user effects of ECs. Some of the reviewed EC studies used more than one type of assessment method. However, none of these identified articles involved a systematic risk comparison of EC and CC use based on their risk to the user. 

### 2.3. Data Collection

All data used in the assessment were collected by document review. Data related to hazard identification and dose-response relationships were collected from reports or documents from authorities such as the International Agency for Research on Cancer (IARC) and the Agency for Toxic Substances and Disease Registry (ATSDR). The data related to hazard exposure assessment were collected from the two key reviewed studies, undertaken by Goniewicz et al., (EC study) [12] and Counts et al., (CC study) [13]. Each study used a different approach to quantify the hazards associated with EC or CC emissions. In Goniewicz’s study, the hazards in EC emissions were identified and quantified by measuring the toxic compounds extracted from vapours into a solid or liquid phase, and analysed with chromatographic and spectroscopy methods [12]. In Counts’ study, the hazards in CC emissions were identified by analysing the mainstream smoke produced by commercial cigarettes using the International sOrganisation for Standardisation (ISO), the U.S. Massachusetts Department of Public Health (MDPH) and the Health Canada (HC) smoking regimens [13]. 

### 2.4. Data Analysis

Two main data analysis methods were used in this study: risk characterisation and risk comparison. Risk characterisation refers to the analysis and summarising of data collected in each step of the risk assessment. Risk comparison refers to compare the risk of exposure to each identified hazard posed using ECs or CCs, as well as collectively estimating and comparing the health risk of using ECs and CCs. 

## 3. Results

### 3.1. Scope Planning

Six main categories were considered under the rubric of “scope”: the tested nicotine delivery devices, the hazards, the sources of the hazards, the routes of exposure, the health effects, and the target population. Analysis was restricted to two nicotine delivery systems: ECs and CCs. In total, 12 EC products and 50 CC products were assessed in the two key reviewed studies.

Based on our literature review, 12 toxicants in ECs and CCs emissions are regarded as the most significant hazards. These 12 toxicants were selected for assessment. Of these, acetaldehyde (Ace), acrolein (Acr) and formaldehyde (Frm) are chemicals from the carbonyl compounds group [12]. Diethylene glycol (DEG) and propylene glycol (PG) are from the diols group. Arsenic (As), cadmium (Cd), chromium (Cr) and nickel (Ni) are heavy metals [14]. Carbon monoxide (CO) is found in CC emissions [15], and is a toxic gas. Finally, *N*′-nitrosonornicotine (NNN) and 4-(methylnitrosamino)-1-(3-pyridyl)-1-butanone (NNK) are tobacco-specific nitrosamines (TSNAs).

The emissions of the tested nicotine delivery devices were considered to be the major sources of hazards of ECs and CCs [16,17] with inhalation being the sole route of exposure [18,19]. The scope of the health impact was restricted to the categories of cancer, cardiovascular diseases (CVD) and respiratory diseases as these are considered the greatest health issues associated with tobacco use as reported by the United States Centres for Disease Control and Prevention (U.S. CDC) [20].

The estimates of exposure are based on current smokers, defined as people having smoked more than 100 cigarettes in their lifetime and at least one cigarette in the last 28 days [21]. The average daily CC consumption of current New Zealand (NZ) smokers is 11 CCs per day [21,22]. Goniewicz and colleagues equate one CC smoked to one EC vaping session consisting of 15 puffs, making the average daily EC consumption of NZ current smokers 11 vaping sessions (15 puffs/session × 11 sessions = 165 puffs) [12]. 

### 3.2. Hazard Identification

In this step, toxicokinetic and toxicodynamic data were collected from the literature for each chemical identified.

#### 3.2.1. Toxicokinetics

Most of the identified hazards are typically absorbed by inhalation, although PG and DEG are usually found in products that are ingested [23,24]. All identified hazards can travel to different parts of the body once absorbed. There is limited information about where inhaled DEG is distributed in the human body [23]. Some metabolites are harmful while some do not pose a significant health risk. Cd and Ni usually accumulate instead of going through any specific metabolism processes [25,26]. Most identified hazards are excreted by the renal system. Some hazards (Acr, Frm, As, and Cd) are excreted via multiple pathways (renally, by defecation or by exhalation) [27,28,29,30]. CO is excreted by exhalation [15].

#### 3.2.2. Toxicodynamics

All identified hazards may contribute to different types of adverse respiratory outcomes. Only CO and Cd have been identified as having a significant impact on the cardiovascular system [18,25].

#### 3.2.3. Carcinogenicity Classification

The carcinogenicity classification of the identified hazards is based on the IARC monograph [31]. Of the 12 identified chemicals, seven (Frm, As, Cd, Cr, Ni, NNK and NNN) are classified as Group 1 carcinogens (carcinogenic to humans), one (Ace) is a Group 2B carcinogen (possibly carcinogenic to humans), one (Acr) is a Group 3 agent (not classifiable as to its carcinogenicity to humans), and the rest (DEG, PG, CO) are not classified as they are not considered to be carcinogenic [31].

#### 3.2.4. Detection in Tested Devices

Nine out of the 12 hazards of concern have been detected in the emissions of ECs: Ace, Acr, Frm, DEG, PG, Cd, Ni, NNK, and NNN. For CCs, eight out of the twelve hazards of concern were detected in the emissions of CCs: Ace, Acr, Frm, As, Cd, CO, NNK, and NNN.

### 3.3. Dose-Response Assessment

Due to practical concerns and the recommendation of the U.S. EPA risk assessment guideline [11], both linear and non-linear dose-response assessments are performed to evaluate identified carcinogenic hazards, and a non-linear dose response assessment only performed on those identified as non-carcinogenic hazards. Table 1 presents the findings of the dose-response assessment based on the reports/documents of the identified hazards.

Table 2 presents the guideline levels of the identified hazards below which the exposure is not considered a significant health risk for an exposed adult. 

### 3.4. Exposure Assessment

In this study, the frequency of exposure was assumed to be 165 puffs for ECs and 11 CCs per day (equivalent to the NZ daily average consumption amongst current smokers); the duration of exposure is assumed to be one year [11]. The data considered in the exposure assessment are based on two key studies that were reviewed (as noted in Section 2.3).

Based on these two key studies, nine (Ace, Acr, Frm, DEG, PG, Cd, Ni, NNK and NNN) out of the 12 identified hazards were mentioned in relation to EC emissions. DEG is a special case. DEG was not identified by the key reviewed studies (as noted in Section 2.3), but was identified in testing by the U.S. Food and Drug Administration (U.S. FDA). Referring to the test results, one of the 18 tested ECs was found to have 1% DEG content in its liquid [42]. Another toxicant, PG, was also not identified by the key reviewed studies (as noted in Section 2.3), but was identified in the study done by Geiss et al. In Geiss’ study, the hazards in EC emissions were measured by analysing the chemical composition of EC mainstream vapour on simulated indoor environments under controlled conditions using a 30 m^3^ emission chamber [43]. Referring to the study results of 12 tested ECs, the average PG exposure level in ECs emissions is 12.12 mg per vaping session (133.28 mg in 11 vaping sessions) and the maximum exposure level is 12.90 mg per vaping session (141.90 mg in 11 vaping sessions). 

As and Cr were both detected in levels lower than those considered to pose a significant risk. Therefore, for the purpose of this study, they were regarded as not being significant hazards associated with the use of ECs. Since ECs are designed to deliver nicotine without combustion, neither of the reviewed EC studies took CO into consideration when assessing the hazards associated with EC emissions.

On the other hand, ten (Ace, Acr, Frm, As, Cd, CO, NNK and NNN) hazards were identified in CC emissions mentioned by the two key studies. Because the analytical limits of Cr and Ni were not revealed, the exposure levels of Cr and Ni in CC emissions are simply indicated as being “below the analytical limit”.

PG and DEG are not identified as hazards in CC emissions. This is because CCs deliver nicotine through the burning of tobacco leaf, while ECs heat a solution potentially containing PG and DEG. Therefore, PG and DEG are unlikely to be detected in CC emissions.

### 3.5. Risk Characterisation 

Based on the findings above, using ECs exposes users to nine out of the 12 hazards identified as being of concern in CCs. Five of them are Group 1 carcinogens (Frm, Cd, Ni, NNK, and NNN), one is a Group 2B carcinogen (Ace), one is a Group 3 carcinogen (Acr), and two are non-carcinogens (DEG and PG). These nine hazards have been correlated to two major types of health effects: cancer and respiratory effects [20,26,27,28,30,31,32,39].

Assuming the average 11 vaping sessions per day of ECs, exposures for four (Acr, DEG, PG, and Cd) out of the nine hazards reach the maximum safe exposure levels. These hazards’ maximum exposure levels are from 1.2 times to 170 times higher than the corresponding guideline levels. 

CCs expose users to eight out of twelve hazards identified as of concern; five are Group 1 carcinogens (Frm, As, Cd, NNK, and NNN), one is a Group 2B carcinogen (Ace), one is a Group 3 carcinogen (Acr), and one is a non-carcinogen (CO). These eight hazards are linked to all three major types of adverse health: cancer, respiratory effects and cardiovascular effects. Assuming a daily consumption of 11 cigarettes, all identified chemicals’ maximum exposure levels (Ace, Acr, Frm, As, Cd, CO, NNK and NNN) would exceed the guideline levels by factors ranging from 1.58 to 4500.

### 3.6. Risk Comparison

**Figure 1:**
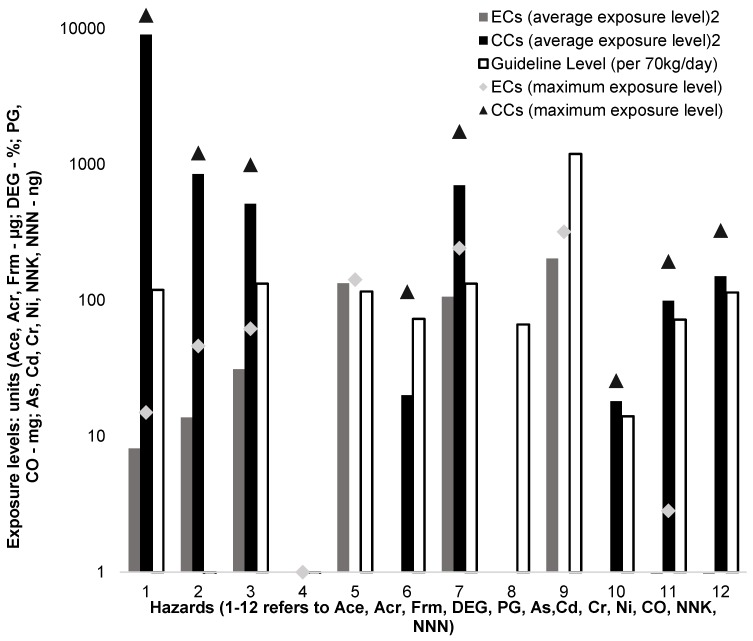
Risk comparison of maximum/average hazards’ exposure levels to guideline levels.

This step in the process uses standardised values to represent the health risk of using the tested nicotine delivery devices being compared. It includes: (1) risk comparison of EC and CC risk profiles, (2) risk comparison of maximum hazard exposure levels (Figure 1), and (3) risk comparison of average hazard exposure levels (Figure 1). 

Based on a risk comparison, using ECs exposes users to nine hazards (seven of them classified as carcinogens) and two are considered to result in adverse health effects. In comparison, using CCs results in exposure to eight hazards (seven of which are carcinogens) and three at levels considered to leads to adverse health effects. The maximum exposure levels to the four hazards found in EC emissions are higher than the guideline levels. In comparison, the maximum exposure levels to the seven hazards in CC emissions are higher than the guideline levels. The average emissions are higher than the guideline level for two of the hazards in EC emissions and seven of the hazards in CC emissions.

## 4. Discussion

Our findings provide evidence that supports the Public Health England statement but was arrived at by applying a different methodology. Although we are aware that some smokers had been smoking for decades, the main purpose of this study is to explore whether they will be exposed to a lower risk of harm by changing to vaping. Hence, no matter how long a smoker had smoked, the probable benefits of changing his/her source of nicotine consumption from CCs to ECs for a year should be very similar. This study leads to two conclusions: that the use of ECs presents a lower risk to health than the use of CCs, and that ECs are likely to be of low health risk to the user.

These results must be interpreted with caution: firstly, CCs have been used for more than a century and there are many more CC users than EC users worldwide. Therefore, far more information is available about the harms of CCs in regard to all criteria listed in the MCDA model. In contrast, ECs are very new products with far fewer users and have limited data available on long-term health consequences. This makes it difficult to assign scores to ECs in relation to criteria, such as product-specific mortality/morbidity and product-related mortality/morbidity. Therefore, the scores for ECs are likely to be less accurate and more biased towards lower risk levels than for CCs. Secondly, there were a number assumptions in our analyses: The daily consumption of CCs and ECs was assumed to be 11 cigarettes or a corresponding 11 vaping sessions over one year; the EC and CC usage patterns in the reviewed studies were assumed to reflect real world use patterns [12,43]; all ECs and CCs were assumed to be similar in their ability to expose users to various hazards and to those tested in the reviewed studies; fourthly, we restricted our assessment to only 12 hazards and three groups of adverse health effects associated with exposure. 

## 5. Conclusions

### 5.1. Implications

The findings of this study have different implications for different groups of people. For smokers, quitting smoking altogether is very likely to lead to great health benefits. However, ECs could be a safer substitute for CCs for those in the process of tobacco cessation. 

For current EC users, it is important to be aware that using some low quality EC products may expose them to a higher risk of harm. EC brands and products with more rigorous quality authentication should be selected. Currently, regulations on EC manufacturing standards and quality are few and far between. Standards for ECs quality and contents should be introduced to prevent low-quality ECs from being sold.

Some health professionals are unclear whether they should recommend ECs to their patients who are smokers. For those patients unable to fully quit smoking or those who refuse to use approved nicotine replacement therapy, ECs could be recommended as an option to reduce exposure to the hazards in CC smoke [16].

### 5.2. Further Research

Standardised methods for assessing EC exposures are needed. By setting standard EC usage patterns (e.g., puff volume, puff frequency, puff duration, puff number per session and so on) and standard EC hazard exposure assessments (e.g., hazard detection limits), EC hazard exposure levels could be compared between different studies. In addition, by setting detection limits in smoking/vaping simulation tests, the hazard exposure levels in EC or CC emissions could be determined more accurately.

### 5.3. Concluding Statement

The health effects of using ECs are still not well understood, but current evidence points to ECs being less harmful than CCs. Using ECs to replace CCs as nicotine delivery products could lead to millions of lives saved and significant reductions in the burden of many smoking-related diseases.

## Figures and Tables

**Table 1:** Findings from the dose-response assessment for the major toxicants in electronic cigarettes (ECs) or conventional cigarettes (CCs).

Hazard	Cancer Risk	Source Agency	Threshold Level	Source Agency
Acetaldehyde	IUCR = 2.2 × 10^−6^	IRIS [32]	CIE (RfC) = 9 × 10^−3^ mg/m^3^	IRIS [32]
Acrolein			CIE (RfC) = 2 × 10^−5^ mg/m^3^	IRIS [33]
Formaldehyde	IUCR = 1.3 × 10^−5^	IRIS [34]	CIE (MRL) = 9.8 × 10^−3^ mg/m^3^	ATSDR [35]
DEG			RfD = 21 mg/70 kg adult/day	SCCP [23]
PG			CIE (RfD) = 116 mg/70 kg adult/day	U.S. EPA [24]
As	IUCR = 4.3 × 10^−3^	IRIS [36]	CIE = 0.0055 μg/m^3^	MFE NZ
Cd	IUCR = 1.8 × 10^−3^	IRIS [25]	CIE (MRL) = 10 ng/m^3^	ATSDR [35]
Cr (VI)	IUCR = 1.2 × 10^−2^	IRIS [37]	CIE (RfC) = 0.0001 mg/m^3^	IRIS [37]
Ni	IUCR = 2.4 × 10^−4^	IRIS [26]	CIE (MRL) = 90 ng/m^3^	ATSDR [35]
CO			15 min RfC = 100 mg/m^3^	WHO [18]
NNK	IUCR = 1.43 × 10^−3^	CEPA [38]	RL < 72 ng per mg	WHO [39]
NNN	IUCR = 4 × 10^−4^	CEPA [40]	RL < 114 ng per mg	WHO [39]

IUCR: Inhalation unit cancer risk (based on lifetime exposure, per μg/m^3^ of identified chemical increase x amount of cancer risk); IRIS: Integrated Risk Information System; CIE: Chronic Inhalation Exposure (≥365 days); RfC: Reference Concentration; MRL: Minimal Risk Level; ATSDR: Agency for Toxic Substances and Disease Registry; RfD: Reference Dosage; SCCP: Scientific Committee on Consumer Products; MFE NZ: Ministry for the Environment, New Zealand; CEPA: California Environmental Protection Agency; RL: Regulation Level; WHO: World Health Organisation; DEG: Diethylene glycol; PG: propylene glycol; NNK: 4-(methylnitrosamino)-1-(3-pyridyl)-1-butanone; NNN: *N*′-nitrosonornicotine.

**Table 2:** Guideline levels of identified hazards.

Hazards	Guideline Levels	Source Agency
Acetaldehyde	CIE RfD = 119.25 μg/70 kg/day	IRIS [32]
Acrolein	CIE RfD = 0.27 μg/70 kg/day	IRIS [33]
Formaldehyde	CIE RfD = 132.50 μg/70 kg/day	ATSDR [35]
DEG	<0.1% as e-liquid impurities	SCCP [23]
PG	CIE RfD = 116.00 mg/70 kg/day	US EPA [24]
As	CIE RfD = 72.88 ng/70 kg/day	MFE NZ [41]
Cd	CIE RfD = 132.50 ng/70 kg/day	ATSDR [35]
Cr	CIE RfD = 66.25 ng/70 kg/day	ATSDR [35]
Ni	CIE RfD = 1192.50 ng/70 kg/day	ATSDR [35]
CO	15 min RfD = 14.00 mg/70 kg for 15 min	WHO [18]
NNK	72.00 ng/cigarette or 72.00 ng/vaping session	WHO [39]
NNN	114.00 ng/cigarette or 114.00 ng/vaping session	WHO [39]

Some hazards’ guideline levels were converted by the equation “RfD = (RfC × IR × EF)/BW”; RfD: Reference Dosage (mg/kg/day); RfC: Reference Concentration (mg/m^3^); IR: Intake Rate (m^3^/day) = target population average air intake rate 13.25 m^3^/day; EF: Exposure Factor (unitless) = 1 (only consider inhalation as the exposure route); BW: Body Weight (kg) = assumed target population average body weight 70 kg.
